# Evaluation of Serum/Urine Genomic and Metabolomic Profiles to Improve the Adherence to Sildenafil Therapy in Patients with Erectile Dysfunction

**DOI:** 10.3389/fphar.2020.602369

**Published:** 2020-12-10

**Authors:** Maria Santa Rocca, Alessia Vignoli, Leonardo Tenori, Marco Ghezzi, Maurizio De Rocco Ponce, Giannis Vatsellas, Dimitris Thanos, Roberto Padrini, Carlo Foresta, Luca De Toni

**Affiliations:** ^1^ Unit of Andrology and Reproduction Medicine—Department of Medicine, University of Padova, Padova, Italy; ^2^ Consorzio Interuniversitario Risonanze Magnetiche di Metallo Proteine (CIRMMP), Sesto Fiorentino, Italy; ^3^ Department of Chemistry “Ugo Schiff”, University of Florence, Sesto Fiorentino, Italy; ^4^ Autònoma de Barcelona, Instituto de Investigaciones Biomédicas Sant Pau, Barcelona, Spain; ^5^ Biomedical Research Foundation Academy of Athens (BRFAA), Athens, Greece; ^6^ Department of Medicine, University of Padova, Padova, Italy

**Keywords:** drug safety, adherence, adverse drug reactions, translational research, nuclear magnetic resonance

## Abstract

Type V-phosphodiesterase-inhibitors (PDE5i) are the first choice drugs in the treatment of erectile dysfunction (ED), being effective in 60–70% of patients. However, approximately 50% of patients per year discontinue the treatment with PDE5i after reporting poor drug efficacy or major adverse drug reactions (ADR). To identify early markers of efficacy/safety for the treatment of ED with PDE5i, the basal clinical characteristics of patients, integrated with metabolomics analysis of serum and urine and genomic data, were here correlated with the PDE5i efficacy and the occurrence of ADR upon administration. Thirty-six males with new diagnosis of ED were consecutively recruited and characterized at baseline for anthropometrics, blood pressure, blood glucose, lipid profile, serum levels of thyroid/sex hormones and erectile function evaluated by IIEF-15 questionnaire. Targeted Next Generation Sequencing (NGS) was applied to genes involved in PDE5i pharmacodynamics and pharmacokinetics. Fasting metabolic profiles of serum and urine were assessed by nuclear magnetic resonance (NMR)-based metabolomics analysis. Patients were prescribed on-demand therapy with Sildenafil oro-dispersible film and followed-up after 3 months from recruitment. Baseline data were compared with IIEF-15 score at follow-up and with the occurrence of ADR recorded by a dedicated questionnaire. Twenty-eight patients were finally included in the analysis. Serum LDL-cholesterol levels were increased in those reporting ADR (143.3 ± 13.2 mg/dl ADR vs. 133.1 ± 12.4 mg/dl No ADR; *p* = 0.046). NGS data showed that specific variants of *PDE11A* and *CYP2D7* genes were more represented in drug responders (both relative risk = 2.7 [0.9–5.1]; *p* = 0.04). NMR-based metabolomics showed the highest association between serum LDL-cholesterol metabolites and the occurrence of ADR (Hazard ratio = 17.5; *p* = 0.019). The association between lipid profile and the ADR pattern suggests major cues in the tailoring of ED therapy with PDE5i.

## Introduction

Therapy discontinuation represents a major public health problem. Between 25 and 50% of patients worldwide do not follow prescribed therapies, resulting in significant health and economic issues (van Dulmen et al., 2007). In the USA, suboptimal adherence has been associated with 125,000 deaths, 10% of hospitalizations, and costs up to US$ 289 billion annually (Osterberg and Blaschke, 2005). However, no shared or high impact solutions have been identified so far (Nieuwlaat et al., 2014). A feasible strategy relies on precision medicine, such as the identification of tailored solutions addressing a patient’s specific adherence barrier and the subsequent scale-up to the population level (Zullig et al., 2018).

A suitable application of these concepts is represented by the model of type V-phosphodiesterase (PDE5) inhibitors (PDE5i), representing the first choice therapy for the erectile dysfunction (ED). ED, namely the inability to achieve and/or maintain an adequate degree of erection for a complete sex intercourse, depends on the impaired production of nitric oxide (NO) by the endothelium of cavernous vessels because of an underlying atherosclerotic process. This pattern can be pharmacologically overcome by the use of selective inhibitors of cGMP-dependent PDE5, which are highly expressed in cavernous endothelium, in order to prolong the cGMP half-life and to enhance the residual NO-mediated vasodilating function (Hawksworth and Burnett, 2015). The estimated prevalence of ED worldwide varies from 3 to 76.5% according to age range and, despite it is not considered a life threatening condition, it is associated with important health issues, such as reduced self-esteem and reduced quality of life (Yafi et al., 2016). The use “on-demand” of PDE5i is demonstrated to be effective in 60–70% of patients (Yafi et al., 2016). However, high rates of therapy discontinuation have been reported by several studies, accounting for approximately 50% of patients/year. The reasons for the discontinuation are mainly related to the lack of efficacy and serious side effects (Corona et al., 2016). Thus the identification of early/predictive markers of poor drug efficacy/safety profile for PDE5i may represent a key strategy to improve the treatment of ED and related comorbidities.

The molecular bases underlying the unfavorable efficacy/safety profile of PDE5i are currently under-investigated. Available studies are focused on the pharmacogenetic implications in ED, identifying a panel of genes, related to NO and cGMP pathway, presenting genetic variants that are associated with poor response to PDE5i. However, clinical data on the prognostic value of these markers are not yet available (Lacchini and Tanus-Santos, 2014). In addition, the onset of side effects after PDE5i dosing has been classically ascribed to the inhibitory side-effect on other PDE activities, such as PDE1, 2, 4, 6, and 11. However, this hypothesis is mainly supported by *in vitro* studies (Bischoff, 2004; Taylor et al., 2009). In this context, metabolomics is considered as a novel and valuable tool to characterize the clinical traits associated with drug response. Performed in an unbiased manner, association of metabolomics to systems biology has provided insight into gene networks, metabolic changes, and genotype/phenotype correlations (Widmann et al., 2013).

In this pilot study we applied unbiased metabolomics analysis of serum and urine to integrate genomic data in order to identify markers of unfavorable efficacy/safety profile of the therapy with PDE5i. To this aim, the genetic profile of a group of patients affected by ED was obtained by gene panel-sequencing including genes involved in PDE5i pharmacodynamics and drug metabolism. Finally, genetic data and the metabolic profile of serum and urine at baseline were combined in order to correlate all findings with the subsequent response to PDE5i administration in terms of drug efficacy and adverse drug reactions.

## Methods

### Patients

This single-center, prospective, pathophysiological-genetic study was conducted in agreement with the declaration of Helsinki (Issue Information-Declaration of Helsinki, 2018) and was approved by the Ethic Committee for Clinical Trials of the University-Hospital of Padova (Italy) with protocol number 3982/AO/16 and subsequent amendments.

The study involved the recruitment of male patients with new diagnosis of ED, eligible for treatment with PDE5i, attending the Unit of Andrology and Reproductive Medicine of the University-Hospital of Padova (Italy). Eligibility criteria for the study were: age between 25 and 60 years old, diagnosis of ED with low or intermediate cardiovascular risk level assessed according to the cardiovascular risk charts of the III Princeton Consensus Conference (Nehra et al., 2012), body mass index (BMI) between 19 and 40 kg/m^2^, and the signing of the informed consent to participate in the study. Exclusion criteria were chronic diseases due to intestinal malabsorption, major endocrine disorders, renal or hepatic failure, severe neoplasms, or the presence of psychiatric conditions impeding the participation in the study.

A total of 36 patients were consecutively recruited during the baseline outpatient evaluation from April 2017 to June 2019. Eight of these patients were excluded from further analyses since they missed the follow-up visit. Each patient was evaluated for the clinical history, concomitant drug therapy, anthropometric parameters (weight, height, BMI), blood pressure, blood glucose and lipid profile, serum levels of thyroid-stimulating hormone (TSH), total testosterone (T), luteinizing hormone (LH), and prolactin. The presence of diabetes was diagnosed by fasting blood levels of glucose ≥126 mg/dl or the use of glycemic lowering agents. The presence of dyslipidemia was diagnosed for blood LDL levels ≥ 130 mg/dl or the use of lipidemia lowering agents. The presence of hypogonadism was diagnosed for serum T levels ≤ 10.4 nmol/L. The erectile function was assessed by the administration of the International Index of the Erectile Function 15 questions-validated questionnaire (IIEF-15), and the cardiovascular risk was stratified according to the international guidelines (Nehra et al., 2012). The IIEF-15 comprises a series of question focused, respectively, on the erectile function (questions 1–5 and 15; IIEF-15_ED_), the quality of the orgasm (questions 9 and 10; IIEF-15_ORGASM_), on the sexual desire (questions 11 and 12; IIEF-15_DESIRE_), on the sexual satisfaction (questions 6–8; IIEF-15_SATISFACTION_), and on the overall quality of life (questions 13 and 14; IIEF-15_QOL_). The presence of cavernous artery alterations was diagnosed by dynamic Penile Color-Doppler Ultrasound analysis (P-CDU) after intra-cavernous PGE1 injection as previously described (Caretta et al., 2019). Patients with increased intima-media thickness of the cavernous artery wall (i.e., IMT > 0.3 mm) were then defined as affected by organic ED.

To avoid the dispersion of subjects on the basis of the available PDE5i drugs, all patients recruited in the study were prescribed to use Sildenafil orodispersible film (ODF) as part of the standard of care, whose dosage was determined by the physician according to of good clinical practice criteria in the management of ED (Greenberg et al., 2019). Patients were also instructed to take the drug in starving conditions and to hold the ODF under the tongue for 15 min without the assumption of water, followed by swallowing as previously described (De Toni et al., 2018). This specific dosage was chosen because of its association with an increased drug tolerability profile compared to the generator film coated tablet or standard oral dosing, representing a strategy to ameliorate the patient’s compliance to the therapy and the protocol (De Toni et al., 2018).

At recruitment, and in any case before initiating the on-demand therapy with PDE5i, each patient was requested to provide a sample of peripheral blood for the assessment of genetic screening, which was stored at −80°C until use. Additional samples of blood serum and early morning urine at fasting conditions were obtained for metabolomics assessment. All samples were centrifuged at 1,000 × g for 10 min to remove cell debris and then stored at −80°C until use. All specimens were processed within 1 h from sampling.

For the evaluation of side effects associated with PDEi administration, patients were asked to fill out a questionnaire for the adverse drug reaction (ADR-questionnaire), adapted from the form of the Italian Drug Agency (https://www.aifa.gov.it/moduli-segnalazione-reazioni-avverse). The ADR-questionnaire provided a direct yes/no question about the drug-response and a direct yes/no question about the occurrence of side effects secondary to PDE5i ingestion. In case of a positive answer to the latter question, the patient was asked to answer specific questions regarding the type of the side effect, the duration and the intensity personally experienced, quantified by a score ranging from 0 (very weak) to 5 (strong).

The standard of care of patients with ED includes a follow-up visit after approximately 3 months from basal evaluation. On that occasion, patients enrolled in the study were re-evaluated for the erectile function by the IIEF-15 questionnaire, and the ADR-questionnaire was collected to obtain representative data of the safety-efficacy profile of the therapy with PDE5i. We defined ΔIIEF-15_ED_ as the difference between the score values of IIEF-15 on questions focused on ED, at follow-up and at baseline. Patients reporting the ineffectiveness of the drug, on the basis of their personal experience, were considered as non-responders.

### Targeted Next Generation Sequencing

Genomic DNA was extracted from peripheral blood leucocytes of subjects using QIAamp DNA Blood Mini Kit, according to the manufacturer’s protocol (Qiagen Inc., Hilden, Germany). The quality of the DNA was determined using a NanoDrop-1000 (Thermo Fisher Scientific Inc, Waltham, MA, USA) and Qubit 2.0 fluorometer (Thermo Fisher Scientific Inc, Waltham, MA, USA). A Qubit dsDNA BR (broad range, 2–1,000 ng) Assay Kit and Qubit dsDNA HS (high sensitivity, 0.2–100 ng) Assay Kit were used with a Qubit fluorometer according to the manufacturer’s protocol.

An Agilent SureSelect XT custom library panel for 22 genes (*CYP3A4, CYP2C9, CYP2D6, CYP3A5, NOS1, NOS3, VEGFA, ACE, GNB3, PDE1A, PDE1B, PDE1C, PDE2A, PDE3A, PDE3B, PDE4A, PDE5A, PDE6A, PDE7A, PDE9A, PDE10A, PDE11A*) was designed with the Agilent SureDesign software. For a total Region of interest (ROI) size of 66.183 kb, 2.747 probes were designed, covering in total 83.156 kb.

Targeted NGS library preparation was carried out with the Agilent SureSelect QXT library prep kit and the libraries were then loaded on a 500-cycle (2 × 250 paired ends) reagent cartridge (Illumina, San Diego, CA, USA) and run on a MiSeq sequencer (Illumina, San Diego, CA, USA). Raw sequencing data analysis and variant calling was performed with the Agilent SureCall software using GRCh37/hg19 as reference genome. For each run, average read depth within the ROI was 222X. Of the ROI, 96.5 and 87.2% was covered by at least 50 and 100 reads respectively. PolyPhen-2 (http://genetics.bwh.harvard.edu/pph2/) was used as an *in silico* tool to predict possible impact of non-synonymous variants on the structure and function of the protein.

### NMR Analysis

All serum and urine samples were analyzed via Nuclear Magnetic Resonance (NMR) spectroscopy. One-dimensional ^1^H NMR spectra for all samples were acquired using a Bruker 600 MHz spectrometer (BrukerBioSpin) operating at 600.13 MHz proton Larmor frequency and equipped with a 5 mm PATXI ^1^H-^13^C-^15^N and ^2^H decoupling probe including a *z* axis gradient coil, an automatic tuning-matching (ATM) and an automatic and refrigerate sample changer (SampleJet). A BTO 2000 thermocouple served for temperature stabilization at the level of approximately 0.1 K at the sample. Before measurement, samples were kept for at least 5 min inside the NMR probe head for temperature equilibration (310 K serum, 300 K urine).

Samples were prepared and NMR spectra acquired following the procedures detailed by Vignoli et al. (Vignoli et al., 2019a). For each serum sample, three standard 1D ^1^H NMR spectra namely NOESY, CPMG (selective detection of low molecular weight metabolites), and Diffusion-edited (selective detection of high molecular weight molecules) spectra were acquired. For each urine sample a standard 1D ^1^H NOESY NMR spectrum was acquired. Before applying Fourier transform, raw NMR data were multiplied by an exponential function equivalent to 0.3 Hz line-broadening factor. Phase and baseline distortions were automatically corrected, and then transformed spectra were calibrated (TSP singlet at 0.00 ppm for urine, and glucose doublet at 5.24 ppm for serum) using TopSpin 3.2 (BrukerBiospin).

### Statistical Analysis

Statistical analysis was conducted with SPSS 21.0 for Windows (SPSS, Chicago, IL). The patients were divided on the basis of two categorical endpoints: drug responders/non responders and patients with or without ADR. Fisher’s exact test was used to compare the occurrence of each identified variant between subgroups. Furthermore, differences in continuous variables between subgroups were analyzed by Student’s t test. *p* values < 0.05 were considered as statistically significant.

The correlation between a continuous outcome and a set of continuous variables was evaluated by stepwise regression analysis, while the correlation with a dichotomous outcome was evaluated with a logistic regression analysis.

All 1D NMR spectra, in the range of 0.02–10 ppm, were binned into chemical shift bins of 0.02-ppm. The corresponding spectral areas (obtained using AssureNMR 2.2 software, Bruker BioSpin) were used as input variables for multivariate statistical analysis. The region containing residual water signals (4.5–6.0 ppm) was removed from all datasets. Urine can present large variations due to dilution, thus they were normalized using probabilistic quotient normalization (PQN; Dieterle et al., 2006), whereas serum spectra were not normalized.

All data analyses were performed using the “R” statistical environment (R Core Team, 2014). Multivariate data analyses were performed on processed binned data. Principal Component analysis was used as first exploratory analysis to identify possible outliers. Random Forest (RF) algorithm (Breiman, 2001) was used for classification. RF is a classification algorithm that uses an ensemble of unpruned decision trees (forest), each of which is built on a bootstrap sample of the training data using a randomly selected subset of variables (bins) (Verikas et al., 2011; Touw et al., 2013). The percentage of trees in the forest that assign one sample to a specific class can be inferred as a probability of belonging to a given class (Vignoli et al., 2019b). In our case, each tree was used to predict whether a sample represents a patient that experienced or not adverse effects, or a patient with ED due or not due to organic causes. For all calculations, the R package “Random Forest”4 was used to grow a forest of 3,000 trees, using the default settings.

Quantification of metabolites (in both serum and urine) and lipid fractions (in serum) was performed using the B.I. (Bruker IVDr 2.0.2.) platform (Jiménez et al., 2018). Wilcoxon rank-sum test (Wilcoxon, 1945) was used to infer differences between the metabolite/lipid levels of groups on the biological assumption that metabolite concentrations are not normally distributed. A *p* value < 0.05 was deemed significant. In this pilot study, we chose to consider and discuss results not adjusted for multiple comparisons in order to decrease the risk of missing promising biomarkers. However, we are aware that this could increase the risk of a type I error. Furthermore, for each analyzed variable (metabolites and lipids) the area under the receiver operating characteristics curve (AUROC) was obtained using the R package “caTools.”

The prognostic capacity of the lipid fractions was evaluated calculating logistic regressions, using the standard R function “glm.”

## Results

Of the 36 patients enrolled in the study, eight missed the follow-up visit without giving notice or reasons and were therefore excluded from the analyses. Twenty-eight patients, all of Caucasian ethnicity, were finally included in the analysis and were prescribed to take Sildenafil ODF, in particular Rabestrom^®^ (IBSA Farmaceutici Italia Srl, Lodi, Milan, Italy), the only marketed Sildenafil ODF formulation available on the market at the time of the study. The demographic clinical characteristics of patients at baseline are reported in Table 1.

**TABLE 1:** Basal demographic and clinical parameters of patients with erectile dysfunction included in the group, distinguished for response to type five phosphodiesterase inhibitors and related adverse drug reactions.

		Response to PDE5i		Adverse drug reaction	
Parameter	All patients (N = 28)	Responders (N = 24)	Non-responders (N = 4)	ADR (N = 12)	NO ADR (N = 16)
*Demographic/Clinical*					
Age (years ± SD)	47.6 ± 13.9	48.8 ± 13.25	40.5 ± 17.9	48.1 ± 10.4	47.2 ± 16.12
Height (cm ± SD)	175.5 ± 8.9	175.5 ± 9.5	175.5 ± 4.5	176.2 ± 6.7	175.1 ± 10.1
Weight (kg ± SD)	81.7 ± 17.3	81.6 ± 17.6	82.8 ± 18.3	78.7 ± 16.4	83.5 ± 18.0
BMI (kg/m^2^ ± SD)	26.4 ± 4.7	26.3 ± 4.5	26.9 ± 5.9	25.2 ± 3.7	27.1 ± 5.2
SBP (mmHg ± SD)	133.2 ± 12.3	131.6 ± 11.4	133.9 ± 12.6	135.0 ± 12.9	132.1 ± 12.9
DBP (mmHg ± SD)	81.8 ± 6.0	80.9 ± 5.4	82.1 ± 5.9	82.5 ± 5.0	81.4 ± 6.9
Glucose (mg/dL ± SD)	102.3 ± 9.8	100.3 ± 8.4	103.1 ± 7.3	94.7 ± 6.8	106.2 ± 9.0
Total Chol (mg/dl ± SD)	185.4 ± 38.9	181.0 ± 38.1	187.9 ± 37.6	187.0 ± 30.2	184.4 ± 39.5
HDL Chol (mg/dl ± SD)	48.7 ± 13.9	49.5 ± 14.6	46.0 ± 12.5	46.0 ± 14.4	50 ± 14.8
LDL Chol (mg/dl ± SD)	143.7 ± 17.1	143.3 ± 25.7	149.9 ± 11.2	143.3 ± 13.2	133.1 ± 12.4*
Triglycerides (mg/dl ± SD)	102.1 ± 47.2	98.4 ± 48.9	108.6 ± 34.4	133.0 ± 38.5	86.7 ± 45.9
Total T (nmol/l)	16.9 ± 8.5	17.5 ± 7.2	15.8 ± 9.5	13.1 ± 7.2	18.0 ± 9.8
LH (IU/ml ± SD)	5.2 ± 3.11	4.0 ± 2.6	5.2 ± 3.4	4.9 ± 3.8	5.0 ± 2.9
Prolactin (ng/ml ± SD)	10.7 ± 0.3	10.5 ± 4.6	12.1 ± 2.4	9.8 ± 3.5	11.3 ± 4.9
Organic ED (n/%)	12/42.9%	11/45.8%	1/25%	5/41.7%	7/43.8%
Diabetes (n/%)	7/25.0%	7/14.8%	0/0%	2/16.7%	5/31.3%
Dyslipidemia (n/%)	10/35.7%	8/33.3%	2/50%	3/25.0%	7/43.8%
Hypogonadism (n/%)	4/14.3	2/8.3%	2/50%	1/8.3%	3/18.8%
Thyroid diseases (n/%)	1/3.6%	1/4.2%	0/0%	0/0%	1/6.3%
*Concomitant Drug Therapy*					
Glycemic lowering agents	3/10.7%	3/12.5%	0/0%	1/8.3%	2/12.5%
Lipid lowering agents	2/7.1%	2/8.3%	0/0%	1/8.3%	1/6.3%
Antihypertension agents	5/17.9%	5/20.8%	0/0%	0/0%	5/31.3%
Antiplatelet/anticoagulants	3/10.7%	3/12.5%	0/0%	0/0%	3/18.75%
Poly-therapy	3/10.7%	3/12.5%	0/0%	0/0%	3/18.75%
*Erectile Function*					
IIEF15_ED_ (Score ± SD)	16.5 ± 6.8	17.1 ± 6.4	13.0 ± 8.4	19.5 ± 6.4	14.5 ± 6.4
IIEF15_ORGASM_ (Score ± SD)	7.9 ± 2.5	7.9 ± 2.2	8.0 ± 4.0	8.2 ± 2.5	7.7 ± 2.5
IIEF15_DESIRE_ (Score ± SD)	6.8 ± 2.1	7.0 ± 1.9	5.5 ± 2.6	7.5 ± 2.4	6.4 ± 1.8
IIEF15_SATISFACTION_ (Score ± SD)	9.3 ± 4.9	9.2 ± 3.8	9.5 ± 10.3	12.3 ± 4.2	7.3 ± 4.3
IIEF15_QOL_ (Score ± SD)	6.2 ± 3.9	6.5 ± 3.7	4.5 ± 5.0	7.3 ± 4.1	5.4 ± 3.7

Abbreviations: PDE5i, type 5 phosphodiesterase inhibitors; ADR, adverse drug reaction; SD, standard deviation; BMI, body mass-index; Chol, cholesterol; HDL, High density-lipoprotein; LDL, Low density-lipoprotein; T, serum testosterone; ED, erectile dysfunction; Poly-therapy, the use of two or more different drug categories; IIEF15, 15-question International Index of Erectile Function; QOL, quality of life.

Significance: * = *p* < 0.05 vs. ADR.

We found that the use of PDE5i for the treatment of ED in the whole study group was associated with a significant increase of the mean IIEF-15_ED_ score (respectively: 16.5 ± 6.8 units at basal *vs* 21.6 ± 6.9 units at follow-up; *p* = 0.007). No significant increase was observed for the specific questions focused on the orgasm, sexual desire, sexual satisfaction, and quality of life.

At the follow-up visit, four out of 28 patients reported no drug response to PDE5i on-demand and, therefore, they were considered as non-responders. Importantly, the mean prescribed drug dosage between responders and non-responders was not statistically different (71.9 ± 38.4 mg vs. 51.2 ± 43.9 mg, respectively; *p* = 0.428), ruling out the posology as a major bias in the outcome. Major concomitant drug therapies were the use of glycemic lowering agents, lipid lowering agents, anti-hypertension agents and antiplatelet/anticoagulants (respectively 3, 2, 5, and 2 out of 28 patients). In addition, 3 out 28 patients reported a poly-therapy with two or more different drug categories. However, non-significant clustering of patients reporting concomitant drug therapies was detected between responders and non-responders.

As expected, the IIEF-15_ED_ score of non-responder patients at follow-up showed no significant increase, compared to baseline (respectively: 13.0 ± 8.4 units at baseline vs. 23.3 ± 7.9 units at follow-up; *p* = 0.125). Differently, responders showed a significant increase of the mean value of IIEF-15_ED_ score (respectively: 17.1 ± 6.4 units at baseline vs. 21.3 ± 6.9 units at follow-up; *p* = 0.045). The comparison of basal clinical characteristics showed non obvious differences between responders and non-responder patients. In addition, the response to PDE5i as a dichotomous parameter was not associated with any of the basal clinical parameters in the logistic regression analysis. Similarly, ΔIIEF-15_ED_ as a continuous variable was not significantly correlated with any of the basal clinical parameter in the stepwise regression analysis.

The collection of the ADR questionnaire at follow-up showed that 12 out of 28 patients reported side effects. In particular, 10 patients had side effects directly related to vasodilation, such as headache, flushing, and tachycardia (respectively 7, 2, and 1 out of 12), whereas two patients reported altered vision. Furthermore, in three out of 12 patients, the side effect was of such an extent to discourage the further drug intake despite reporting an effective response to the drug. Accordingly, patients disclosing the discontinuation of the therapy had a higher score of the side effect intensity compared to those patients that continued the adherence to the therapy (respectively: 3.7 ± 0.6 units vs. 0.7 ± 1.1 units; *p* < 0.001). Considering the majority of vascular side effects, patients reporting ADRs were considered as a whole group in the subsequent analyses.

The comparison of clinical characteristics at baseline showed that patients reporting ADR had higher serum LDL levels (respectively: 143.3 ± 13.2 mg/dl ADR vs. 133.1 ± 12.4 mg/dl No ADR; *p* = 0.046). The presence of ADR as a dichotomous parameter was significantly associated with serum LDL levels at logistic regression analysis (Score = 4.634; *p* = 0.0319). However, this association was not maintained at stepwise regression analysis where ADR intensity was considered as a continuous variable.

Importantly, non-significant clustering of concomitant drug therapies was detected between patients that reported or not ADR, albeit 31.3% of the latter used antihypertensive agents (*p* = 0.0525 *vs* patients with ADR), ruling out a major role of the underlying therapy in the onset of ADR.

### Results of Molecular Analysis

A total of 2,629 genetic variants were detected in the target regions. Of the total identified variants, 2,419 were single nucleotide variants and 210 small insertion/deletions. Additionally, 1,251 mapped within the coding regions. The number of detected variants for each gene included in the panel is reported in Table 2.

**TABLE 2:** The gene panel evaluated in 28 male patients with erectile dysfunction included in the study and the corresponding number of gene variants.

ID Gene	*N*° variants out of 2,629 total variants
CYP2C9	18
CYP2D6	504
CYP2D7	113
CYP3A4	33
CYP3A5	14
GNB3	24
LOC101929829	126
LOC105373764	50
NOS1	115
NOS3	142
PDE10A	91
PDE11A	204
PDE1A	87
PDE1B	49
PDE1C	123
PDE2A	232
PDE3A	79
PDE3B	28
PDE4A	45
PDE5A	94
PDE6A	36
PDE7A	28
PDE9A	165
VEGFA	24
ACE	205

Interestingly, a higher number of variants was observed in PDE5i responders compared to non-responders (*p* < 0.001). In addition, a higher number of variants was observed in patients that did not show dyslipidemia at baseline, compared with those patients showing dyslipidemia (*p* = 0.04).

Finally, Table 3 shows the 8 single nucleotide variants that showed a significant and specific clustering into one of the 4 groups of patients showing a defined outcome: responders, non-responders, reporting ADR, reporting no ADR. In particular, rs10201180 variant of *PDE11A* gene and rs56127449 variant of *CYP2D7* gene were significantly more represented in responders to PDE5i, with an equal relative risk of association of 2.7. On the other hand, rs1980091, rs392565, and rs426907 variants of *PDE2A* gene, together rs7966459 variant of *PDE3A*, were significantly associated to the presence of ADR, with a relative risk of association ranging from 2.7 to 3.2. Interestingly, rs1799853 and rs9332119 variants of *CYP2C9* gene represented protective factors against ADR, being indeed more represented in patients reporting no ADR.

**TABLE 3:** Differential clustering of gene variants detected in 28 male patients with erectile dysfunction included in the study, grouped according to drug response of presence of adverse drug reaction.

ID target	SNP	Responders	Non-responders	ADR	NO ADR	Variant
PDE2A	rs1980091			*p* = 0.04; RR = 2.7 [0.9–5.0]		Synonymous variant
PDE2A	rs392565			*p* = 0.02; RR = 3.2 [1.1–4.6]		Synonymous variant
PDE2A	rs426907			*p* = 0.02; RR = 3.2 [1.1–4.6]		Intron variant
PDE3A	rs7966459			*p* = 0.04; RR = 2.7 [0.9–5.0]		Intron variant
PDE11A	rs10201180	*p* = 0.04; RR = 2.7 [0.9–5.1]				Splice region variant
CYP2C9	rs1799853				*p* = 0.02.RR = 0.0 [0–1.02]	Missense variant
CYP2C9	rs9332119				*p* = 0.02.RR = 0.0 [0–1.02]	Intron variant
CYP2D7	rs56127449	*p* = 0.04; RR = 2.7 [0.9–5.1]				Missense variant

Abbreviations: DR, drug responsive; NDR, not drug responsive; ADR, adverse drug reactions; NADR, not adverse drug reactions; RR, Relative Risk (95% confidence intervals are reported within squared brackets).

### Evaluation of the Metabolic Profiles by Metabolomics Analysis

#### Principal Component Analysis of Serum and Urine Samples

**FIGURE 1:**
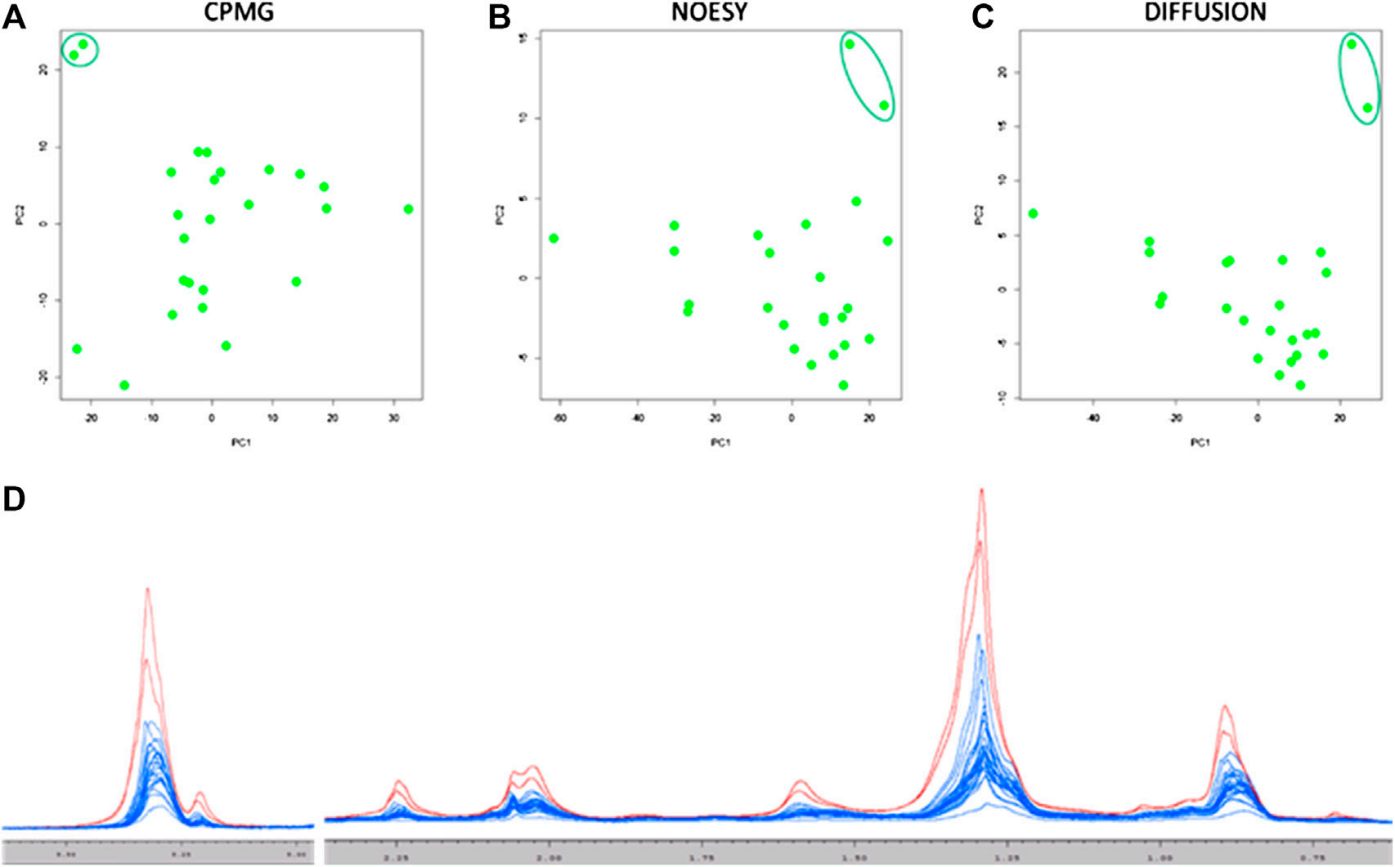
Score plots of the first two principal components of the PCA analysis on the three serum NMR spectra acquired: **(A)** CPMG; **(B)** NOESY; **(C)** Diffusion. All Diffusion spectra are reported in panel **(D)**, the two outlier samples are colored in red (one responder patient and one non-responder patient).

Principal component analysis (PCA) was used as first exploratory analysis. Two serum samples shown to be outliers in all the three kind of NMR spectra acquired (Figures 1A–C). Both serum samples (Figure 1D) had peculiar high levels of lipids and lipoproteins and, interestingly, both patients showed the highest BMI values (BMI > 31). Consequently, both samples were removed from the study group for the following analyses. On the contrary, no evident clustering neither outlier was present in urine and thus, all samples were considered in the modeling.

#### Multivariate Discrimination Between Patients with Erectile Dysfunction Associated with Organic Causes Compared to All Other Causes

**FIGURE 2:**
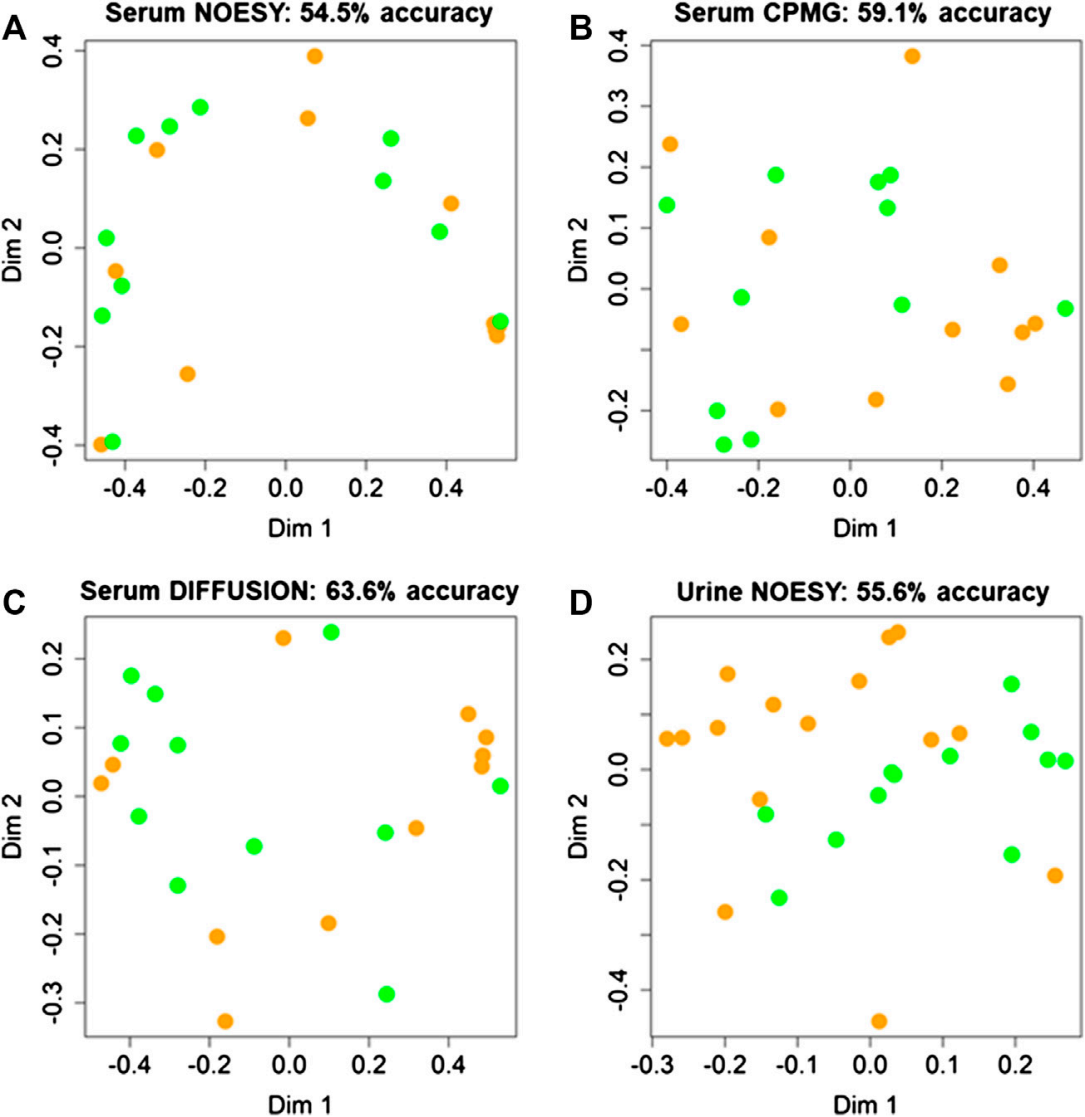
Confusion matrices and score plots of the first two principal components of the Random Forest models discriminating patients with erectile disfunction induced by organic causes (green) and those for whom the disease was related by other causes (orange) calculated on the NMR spectra acquired: **(A)** serum NOESY; **(B)** serum CPMG; **(C)** serum DIFFUSION; **(D)** urine NOESY.

**FIGURE 3:**
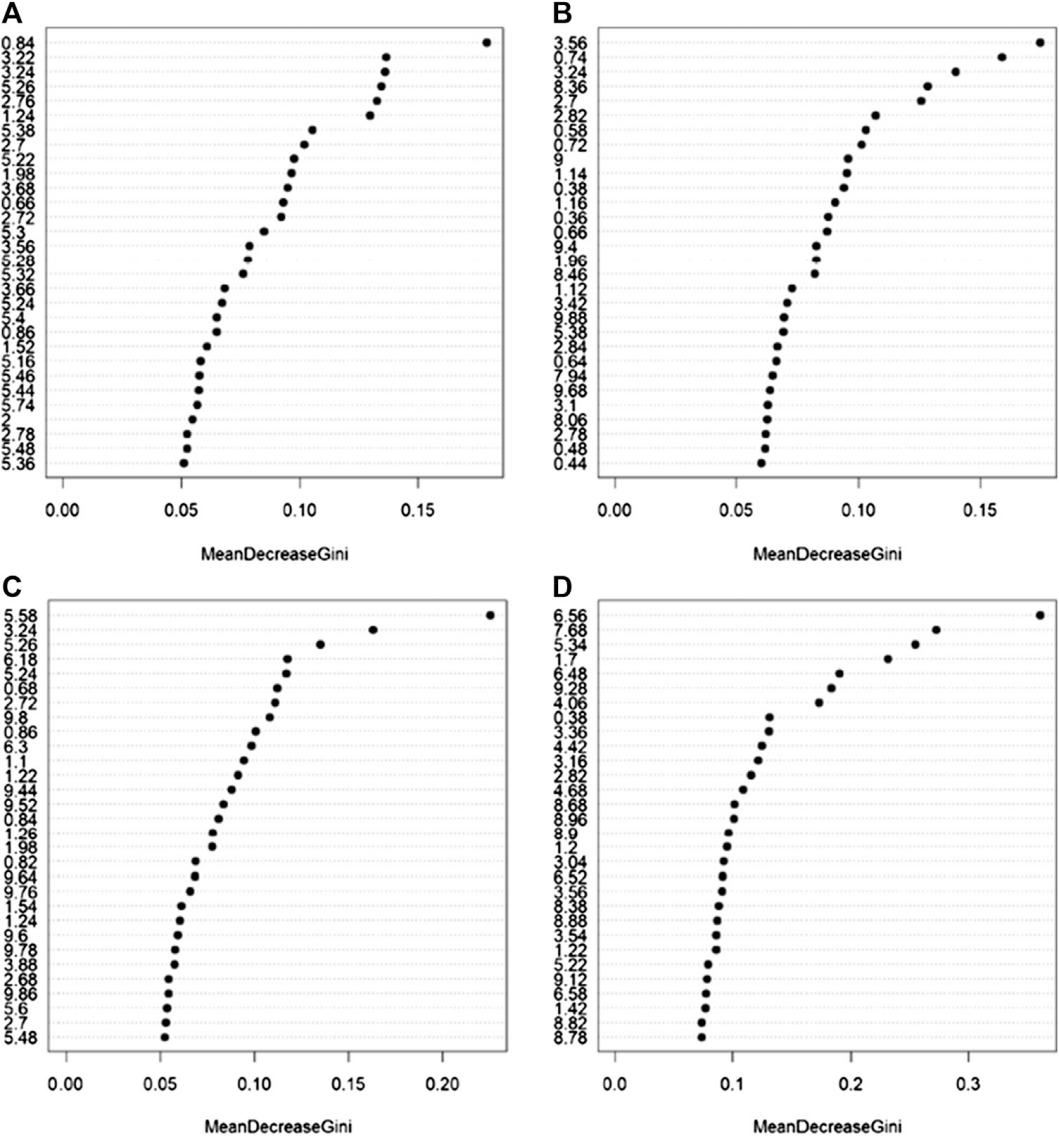
Bins that mainly contribute to the discrimination between patients with erectile dysfunction induced by organic causes and those for whom the disease was related by other causes calculated on the NMR spectra acquired: **(A)** serum NOESY; **(B)** serum CPMG; **(C)** serum DIFFUSION; **(D)** urine NOESY. The importance is measured by the Gini index.

Random Forest models built using serum NOESY (54.5% accuracy, 54.5% sensitivity, 54.5% specificity), CPMG (59.1% accuracy, 54.5% sensitivity, 63.6% specificity), Diffusion (63.6% accuracy, 63.6% sensitivity, 63.6% specificity) spectra, and urine NOESY spectra (55.6% accuracy, 33.3% sensitivity, 73.3% specificity) did not show any obvious difference between patients with ED associated with organic causes and those for whom the disease was related to other causes (Figures 2A–D). The bins that mainly contribute to the group discrimination in each model are reported in Figure 3. These results suggest that the patient’s metabolic profiles, evaluated by metabolomics approach, are not significantly influenced by the disease etiology.

#### Multivariate Discrimination Between Patients That Reported or did not Report Adverse Drug Reactions

**FIGURE 4:**
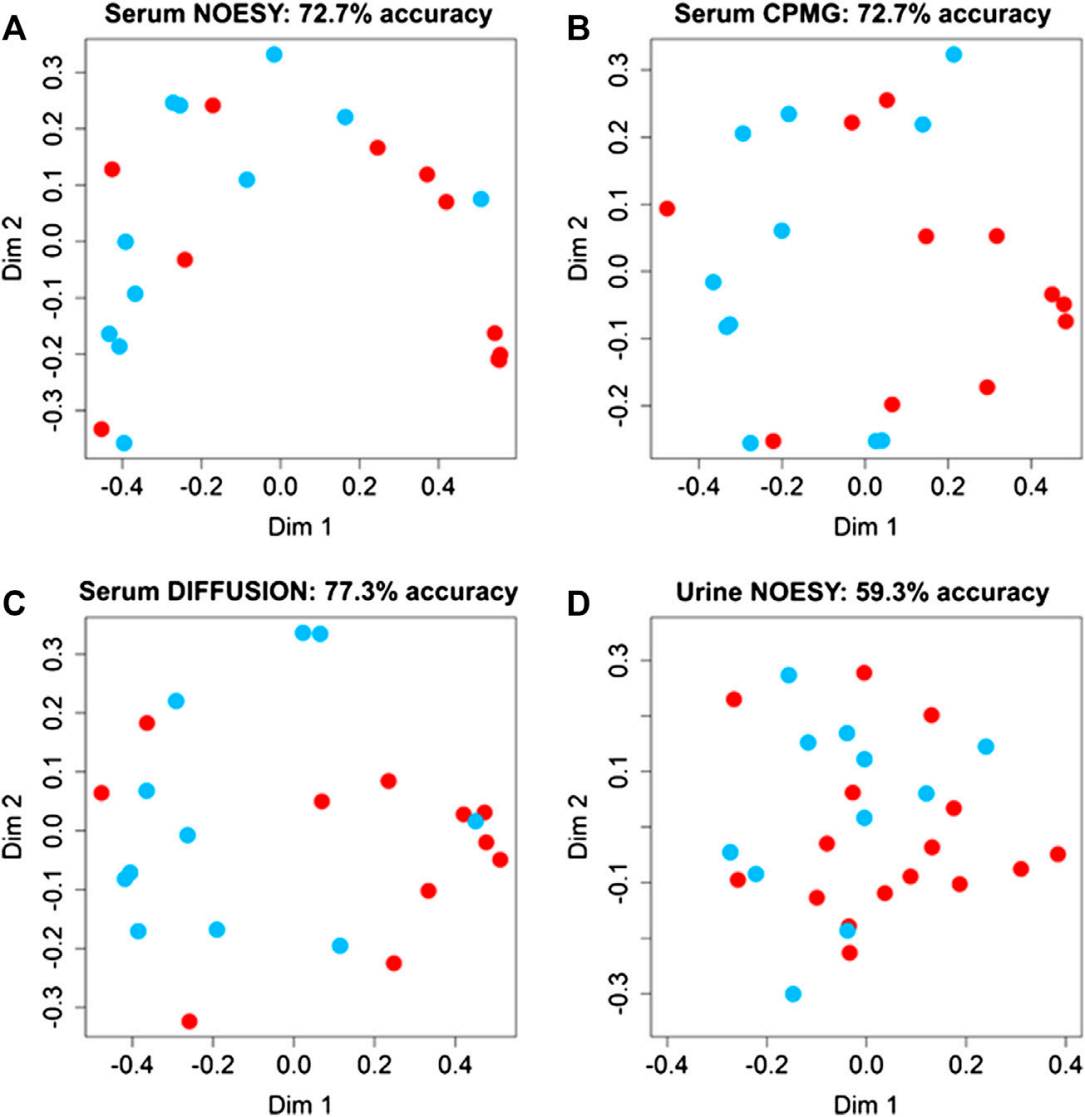
Confusion matrices and score plots of the first two principal components of the Random Forest models discriminating patients that experienced (red dots) and not experienced (blued dots) adverse effects to the administration of PDE5 inhibitors calculated on the NMR spectra acquired: **(A)** serum NOESY; **(B)** serum CPMG; **(C)** serum DIFFUSION; **(D)** urine NOESY.

Random Forest models (Figures 2A–C) built using NOESY (72.7% accuracy, 81.8% sensitivity, 63.6% specificity), CPMG (72.7% accuracy, 72.7% sensitivity, 72.7% specificity), and Diffusion (77.3% accuracy, 81.8% sensitivity, 72.7% specificity) spectra showed statistically significant discrimination accuracy between patients that reported or not ADR after the administration of PDE5 inhibitors. Conversely, only slight discrimination (59.3% accuracy, 36.4% sensitivity, 75.0% specificity) between these two groups was present in the urine analysis (Figure 4D). The bins that mainly contribute to the group discrimination in each model are reported in Supplementary Figure S3.

**FIGURE 5:**
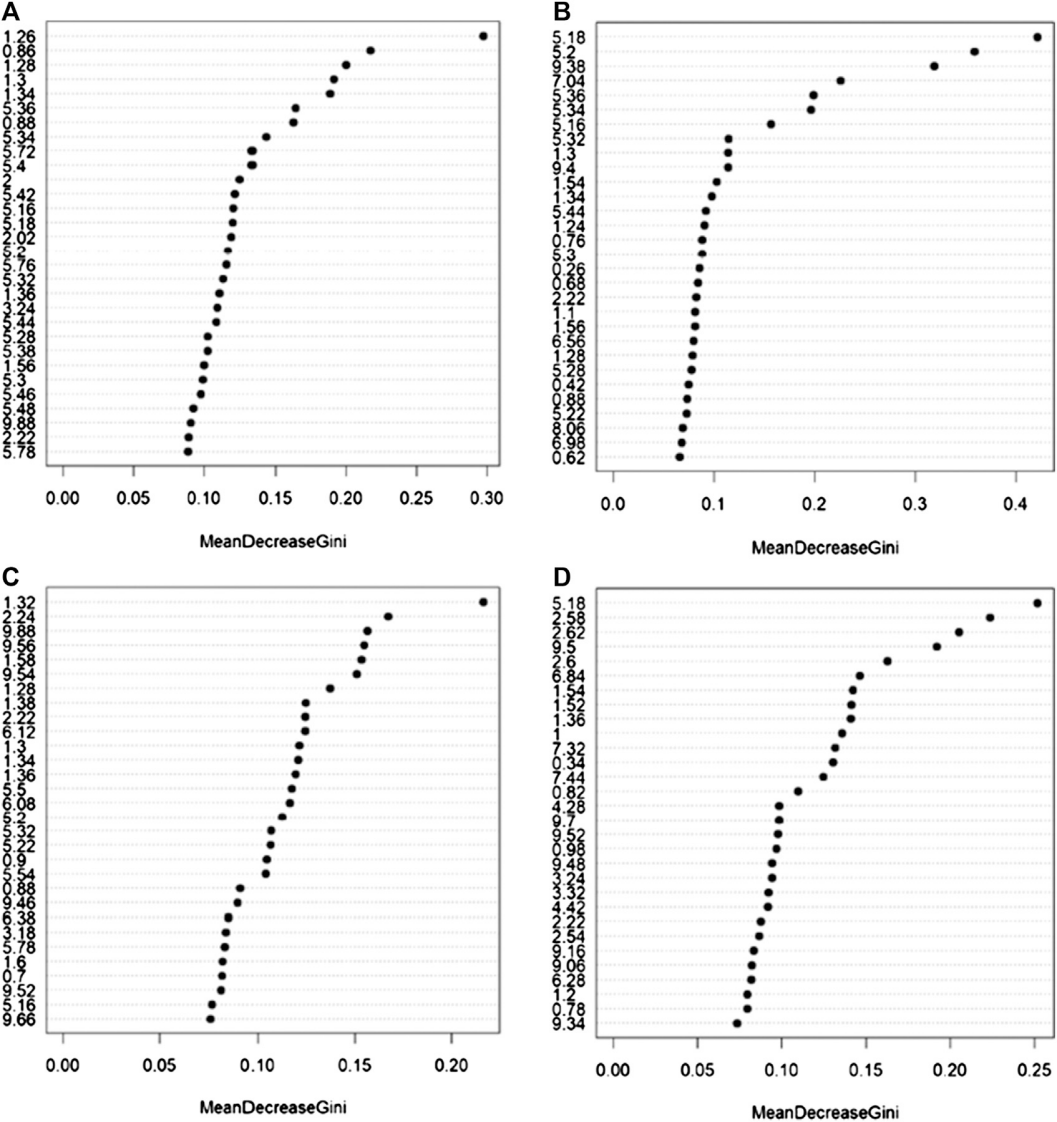
Bins that mainly contribute to the discrimination between patients that experienced and not experienced adverse effects to the administration of PDE5 inhibitors calculated on the NMR spectra acquired: **(A)** serum NOESY; **(B)** serum CPMG; **(C)** serum DIFFUSION; **(D)** urine NOESY. The importance is measured by the Gini index.

#### Univariate Analysis for the Discrimination Between Patients That Reported or did not Report Adverse Drug Reactions

Univariate metabolite analysis enables the identification of several differences between patients that reported or not ADR after the administration of PDE5i in serum lipid fractions. Results are reported in Table 4. Conversely, only the levels of threonine showed statistically significant differences in serum and no significant difference was observed for low molecular weight metabolites in urine (respectively, Tables 5, 6).

**TABLE 4:** Lipid analysis in serum samples from the 26 out of 28 patients with ED included in the metabolomics analysis.

Lipid Fraction	*p*-value	AUROC	Log2(FC)
**Triglycerides (TG)**	**0.019**	**0.793**	**0.422**
**Cholesterol (Chol)**	**0.019**	**0.793**	**0.417**
**LDL Cholesterol (LDL Chol)**	**0.028**	**0.777**	**0.541**
HDL Cholesterol (HDL Chol)	0.401	0.612	0.163
Apo A1	0.193	0.669	0.193
Apo A2	0.212	0.661	0.294
**Apo B100**	**0.040**	**0.760**	**0.480**
**Lipoprotein Main Fractions Triglycerides VLDL**	**0.007**	**0.835**	**0.675**
**Lipoprotein Main Fractions Triglycerides IDL**	**0.023**	**0.785**	**0.797**
Lipoprotein Main Fractions Triglycerides LDL	0.108	0.707	0.349
Lipoprotein Main Fractions Triglycerides HDL	0.974	0.508	0.005
**Lipoprotein Main Fractions Cholesterol VLDL**	**0.023**	**0.785**	**0.590**
**Lipoprotein Main Fractions Cholesterol IDL**	**0.023**	**0.785**	**0.912**
**Lipoprotein Main Fractions Cholesterol LDL**	**0.028**	**0.777**	**0.541**
Lipoprotein Main Fractions Cholesterol HDL	0.401	0.612	0.163
**Lipoprotein Main Fractions Free Cholesterol VLDL**	**0.016**	**0.802**	**0.512**
**Lipoprotein Main Fractions Free Cholesterol IDL**	**0.013**	**0.810**	**0.951**
**Lipoprotein Main Fractions Free Cholesterol LDL**	**0.019**	**0.793**	**0.443**
Lipoprotein Main Fractions Free Cholesterol HDL	0.699	0.554	0.089
**Lipoprotein Main Fractions Phospholipids VLDL**	**0.019**	**0.793**	**0.594**
**Lipoprotein Main Fractions Phospholipids IDL**	**0.015**	**0.810**	**0.927**
**Lipoprotein Main Fractions Phospholipids LDL**	**0.034**	**0.769**	**0.450**
Lipoprotein Main Fractions Phospholipids HDL	0.606	0.570	0.099
Lipoprotein Main Fractions Apo A1 HDL	0.243	0.653	0.180
Lipoprotein Main Fractions Apo A2 HDL	0.243	0.653	0.280
**Lipoprotein Main Fractions Apo B VLDL**	**0.034**	**0.769**	**0.537**
**Lipoprotein Main Fractions Apo B IDL**	**0.023**	**0.785**	**0.742**
**Lipoprotein Main Fractions Apo B LDL**	**0.047**	**0.752**	**0.481**
**VLDL Subfractions Triglycerides VLDL 1**	**0.016**	**0.802**	**0.566**
**VLDL Subfractions Triglycerides VLDL 2**	**0.007**	**0.843**	**0.866**
**VLDL Subfractions Triglycerides VLDL 3**	**0.004**	**0.851**	**0.980**
**VLDL Subfractions Triglycerides VLDL 4**	**0.007**	**0.835**	**0.833**
VLDL Subfractions Triglycerides VLDL 5	0.797	0.537	0.082
VLDL Subfractions Cholesterol VLDL 1	0.438	0.603	0.264
**VLDL Subfractions Cholesterol VLDL 2**	**0.022**	**0.793**	**0.677**
**VLDL Subfractions Cholesterol VLDL 3**	**0.006**	**0.851**	**1.068**
**VLDL Subfractions Cholesterol VLDL 4**	**0.019**	**0.793**	**0.889**
VLDL Subfractions Cholesterol VLDL 5	0.768	0.541	0.048
VLDL Subfractions Free Cholesterol VLDL 1	0.088	0.719	0.721
**VLDL Subfractions Free Cholesterol VLDL 2**	**0.019**	**0.793**	**0.741**
**VLDL Subfractions Free Cholesterol VLDL 3**	**0.009**	**0.835**	**1.058**
**VLDL Subfractions Free Cholesterol VLDL 4**	**0.013**	**0.818**	**1.040**
VLDL Subfractions Free Cholesterol VLDL 5	0.554	0.579	−0.174
VLDL Subfractions Phospholipids VLDL 1	0.047	0.752	0.605
**VLDL Subfractions Phospholipids VLDL 2**	**0.005**	**0.843**	**0.803**
**VLDL Subfractions Phospholipids VLDL 3**	**0.003**	**0.876**	**1.012**
**VLDL Subfractions Phospholipids VLDL 4**	**0.017**	**0.806**	**0.720**
VLDL Subfractions Phospholipids VLDL 5	0.430	0.603	0.134
LDL Subfractions Triglycerides LDL 1	0.652	0.562	0.114
**LDL Subfractions Triglycerides LDL 2**	**0.040**	**0.760**	**0.319**
LDL Subfractions Triglycerides LDL 3	0.622	0.566	0.147
LDL Subfractions Triglycerides LDL 4	0.101	0.711	0.395
**LDL Subfractions Triglycerides LDL 5**	**0.033**	**0.773**	**0.534**
**LDL Subfractions Triglycerides LDL 6**	**0.049**	**0.752**	**0.354**
LDL Subfractions Cholesterol LDL 1	0.300	0.636	0.261
LDL Subfractions Cholesterol LDL 2	0.519	0.587	0.257
LDL Subfractions Cholesterol LDL 3	0.065	0.736	0.481
**LDL Subfractions Cholesterol LDL 4**	**0.019**	**0.793**	**0.590**
**LDL Subfractions Cholesterol LDL 5**	**0.028**	**0.777**	**0.711**
**LDL Subfractions Cholesterol LDL 6**	**0.040**	**0.760**	**0.582**
LDL Subfractions Free Cholesterol LDL 1	0.057	0.744	0.399
LDL Subfractions Free Cholesterol LDL 2	0.171	0.678	0.424
**LDL Subfractions Free Cholesterol LDL 3**	**0.040**	**0.760**	**0.361**
**LDL Subfractions Free Cholesterol LDL 4**	**0.034**	**0.769**	**0.529**
**LDL Subfractions Free Cholesterol LDL 5**	**0.040**	**0.760**	**0.619**
**LDL Subfractions Free Cholesterol LDL 6**	**0.047**	**0.752**	**0.593**
LDL Subfractions Phospholipids LDL 1	0.401	0.612	0.190
LDL Subfractions Phospholipids LDL 2	0.562	0.579	0.225
LDL Subfractions Phospholipids LDL 3	0.065	0.736	0.398
**LDL Subfractions Phospholipids LDL 4**	**0.019**	**0.793**	**0.520**
**LDL Subfractions Phospholipids LDL 5**	**0.023**	**0.785**	**0.654**
**LDL Subfractions Phospholipids LDL 6**	**0.034**	**0.769**	**0.518**
LDL Subfractions Apo B LDL 1	0.293	0.636	0.203
LDL Subfractions Apo B LDL 2	0.401	0.612	0.253
**LDL Subfractions Apo B LDL 3**	**0.040**	**0.760**	**0.427**
**LDL Subfractions Apo B LDL 4**	**0.019**	**0.793**	**0.576**
**LDL Subfractions Apo B LDL 5**	**0.028**	**0.777**	**0.691**
**LDL Subfractions Apo B LDL 6**	**0.047**	**0.752**	**0.522**
HDL Subfractions Triglycerides HDL 1	0.470	0.595	−0.311
HDL Subfractions Triglycerides HDL 2	0.652	0.562	−0.067
HDL Subfractions Triglycerides HDL 3	0.431	0.603	0.159
HDL Subfractions Triglycerides HDL 4	0.148	0.686	0.202
HDL Subfractions Cholesterol HDL 1	0.847	0.529	0.005
HDL Subfractions Cholesterol HDL 2	0.844	0.529	0.108
HDL Subfractions Cholesterol HDL 3	0.438	0.603	0.221
HDL Subfractions Cholesterol HDL 4	0.101	0.711	0.270
HDL Subfractions Free Cholesterol HDL 1	0.949	0.512	0.148
HDL Subfractions Free Cholesterol HDL 2	0.606	0.570	0.223
HDL Subfractions Free Cholesterol HDL 3	0.332	0.628	0.403
HDL Subfractions Free Cholesterol HDL 4	0.082	0.723	0.352
HDL Subfractions Phospholipids HDL 1	0.478	0.595	−0.153
HDL Subfractions Phospholipids HDL 2	0.949	0.512	0.043
HDL Subfractions Phospholipids HDL 3	0.669	0.558	0.193
HDL Subfractions Phospholipids HDL 4	0.171	0.678	0.215
HDL Subfractions Apo A1 HDL 1	0.606	0.570	−0.194
HDL Subfractions Apo A1 HDL 2	0.797	0.537	0.100
HDL Subfractions Apo A1 HDL 3	0.438	0.603	0.176
HDL Subfractions Apo A1 HDL 4	0.076	0.727	0.247
HDL Subfractions Apo A2 HDL 1	0.844	0.529	0.073
HDL Subfractions Apo A2 HDL 2	0.699	0.554	0.328
HDL Subfractions Apo A2 HDL 3	0.401	0.612	0.344
**HDL Subfractions Apo A2 HDL 4**	**0.040**	**0.760**	**0.300**

A positive Log2(FC) value means higher level in patients that experienced adverse drug reactions. Abbreviations: AUROC, Area under the receiver operating characteristics curve. Associations with p values <0.05 are reported in bold.

**TABLE 5:** Serum metabolite univariate analysis.

	*P*-value	AUROC	Log2(FC)
Alanine	0.308	0.632	0.077
Creatinine	0.687	0.554	0.109
Glutamic acid	0.528	0.579	0.145
Glutamine	0.375	0.616	0.133
Glycine	0.717	0.550	0.064
Histidine	0.344	0.620	0.144
Isoleucine	0.612	0.566	0.065
Leucine	0.146	0.686	0.241
Lysine	0.817	0.533	−0.199
Ornithine	0.573	0.574	0.090
Phenylalanine	0.402	0.607	0.215
**Threonine**	**0.009**	**0.831**	**0.621**
Tyrosine	0.893	0.521	0.022
Valine	1.000	0.504	0.040
Acetic acid	0.916	0.517	−0.038
Citric acid	0.895	0.521	0.074
Formic acid	0.503	0.587	0.135
Lactic acid	0.393	0.612	0.020
3-Hydroxybutyric acid	0.099	0.702	−1.728
Acetone	0.588	0.570	0.142
Pyruvic acid	0.387	0.612	−0.096
Glucose	0.278	0.640	0.145

A positive Log2(FC) means higher level in patients that experienced adverse effects. Associations with p values <0.05 are reported in bold.

**TABLE 6:** Metabolite analysis in urine samples.

	*p*-value	AUROC	Log2(FC)
Acetic acid	0.671	0.548	0.940
Acetoacetic acid	0.448	0.557	−1.339
Acetone	0.098	0.685	−1.030
Alanine	0.865	0.523	0.032
Betaine	0.604	0.563	−0.202
Citric acid	0.368	0.608	0.218
Creatinine	0.481	0.585	0.064
D-Glucose	0.249	0.605	1.042
Formic acid	0.276	0.625	−0.428
Glycine	0.336	0.614	−0.242
Hippuric acid	0.486	0.580	0.127
Methanol	0.535	0.571	−0.099
Methylmalonic acid	0.812	0.523	0.113
N-Isovaleroylglycine	0.812	0.523	0.216
N-N-Dimethylglycine	0.297	0.622	−0.739
Oxaloacetic acid	0.059	0.696	−1.742
Oxypurinol	0.120	0.614	3.862
Proline betaine	0.520	0.557	−1.698
Propylene glycol	0.497	0.551	−2.066
Succinic acid	0.110	0.676	−0.968
Tartaric acid	0.314	0.614	−1.097
Trigonelline	0.974	0.506	−0.048
Uracil	0.480	0.580	0.467
Valine	0.361	0.608	0.325

A positive Log2(FC) means higher level in patients that experienced adverse effects.

Since the serum lipid fractions showed the best prognostic values for ADR, hazard ratios (HR) for the seven main lipid fractions (namely triglycerides, total cholesterol, LDL-cholesterol, HDL-cholesterol, ApoA1, ApoA2, ApoB100) were calculated, setting each respective median value as arbitrary threshold. As reported in Table 7, triglycerides, total cholesterol, and LDL-cholesterol showed the most significant association, with LDL-cholesterol having the highest hazard ratio.

**TABLE 7:** Association between the main lipid fractions, obtained by metabolomics analysis, and the occurrence of adverse drug reactions in 26 out of 28 patients with ED included in the analysis.

Main Lipid Fraction	HR	*p*-value
**Triglycerides, TG ≥ 80**	**7.111**	**0.040**
**Cholesterol, Chol ≥ 190**	**7.111**	**0.040**
**LDL Cholesterol, LDL Chol ≥ 100**	**17.500**	**0.019**
HDL Cholesterol, HDL Chol ≥ 50	0.686	0.665
Apo A1, Apo A1 ≥ 140	1.458	0.665
Apo A2, Apo A2 ≥ 30	1.458	0.665
Apo B100, Apo B100 ≥ 80	3.200	0.200

Abbreviations: HR, hazard ratio.

Risk threshold for each lipid fractions were arbitrarily set at each median value. Associations with p values <0.05 are reported in bold.

## Discussion

This is the first study focusing on the early identification of markers of efficacy and safety of PDE5i, used as the first-choice treatment of ED, through the analysis of the clinical phenotype of patients in association with their genetic profile, concerning the genes involved in PDE5i pharmacodynamics and drug metabolism, and the metabolic profile of serum and urine assessed by unbiased metabolomics analysis. We found that the pharmacological response to Sildenafil is essentially related to the genetic background of the individual, particularly to the pattern of variants localized in genes belonging to the PDE family. On the other hand, the occurrence of ADR showed a mixed correlation both with the genetic profile, particularly with genes involved in hepatic drug metabolism, and importantly with the clinical phenotype of the patients, being largely related with the serum LDL-lipid profile.

In this study, the possible genetic base of the efficacy/safety profile PDE5i was assessed through the sequencing of genes involved in pharmacodynamics and pharmacokinetics of this class of drugs. The occurrence of ADR associated with the use of Sildenafil has been classically ascribed to the side inhibitory activity towards other cGMP-dependent phosphodiesterases (Bischoff, 2004). In particular, the expression of PDE3A in the gastrointestinal tissue, cardiovascular system, and brain has been linked with headache, flushing, and dyspepsia associated with the use of PDE5i (Goldstein et al., 1998; Wallis et al., 1999). We found significant clustering of genetic variants rs392565 and rs426907 of *PDE2A* gene and rs7966459 variant of *PDE3A* gene, respectively, in patients reporting ADR, further supporting this model. Interestingly, the rs10201180 variant in *PDE11A* gene, corresponding to a splice region variant, was significantly associated with the responding phenotype in our cohort of ED patients. PDE11A owns a mixed activity both on cAMP and cGMP, and the side inhibition on PDE11 has been typically associated with the back and muscle pain ascribed to the treatment with Tadalafil, another PDE5i (Bischoff, 2004). This evidence deserves further insights, suggesting a possible role of this protein as a co-target in the pharmacodynamic of Sildenafil. On the other hand, the association of the occurrence of ADR with genetic variants of cytochromes involved in drug metabolism appears as novel in regard of the treatment with PDE5i. This is particularly the case of rs1799853, a missense variant of *CYP2C9* gene, predicted to be pathogenic by PolyPhen-2, and previously associated with a reduced drug metabolic function of the CYP2C9 cytochrome (Helin et al., 2019; Calderon-Ospina et al., 2020). Although the potential pharmacokinetic impact of the identified genetic variants on the phenotype goes beyond the aim of this study, this evidence suggests that the occurrence of ADR may rely on individual fluctuations of the PDE5i serum levels due to functional variations of the enzymes involved in drugs metabolism.

Analogous considerations can be drawn from the results of metabolic profiling. To date, few studies have correlated the serum lipid profile with the response to PDE5i with non-conclusive results. In a study performed on 45 patients with ED, Solomon et al. 2006 showed that the acute vascular response to Sildenafil, evaluated by photoplethysmography-based technique, negatively correlated with basal lipoprotein levels (Solomon et al., 2006). Furthermore, a placebo-controlled randomized clinical trial including 118 non-responders to Sildenafil showed that that the adjunct treatment with atorvastatin, resulting in serum cholesterol reduction, was associated with a significant increase of the IIEF-15_ED_ score, particularly in those patients with moderate/severe ED (Dadkhah et al., 2010). Conversely, dyslipidemia did not significantly impact the response to Vardenafil as described by Miner et al. in a Post-hoc subgroup analysis designed to address the influence of serum lipid levels and the presence of metabolic syndrome (Miner et al., 2010). However, neither of the three studies considered the occurrence of ADR in the overall response to PDE5i that, in the present study, was mostly predicted by the elevated serum levels of LDL, as depicted by both clinical data and the untargeted analysis of serum metabolites by NMR. Of note, the energy metabolism phenotype has been shown to significantly alter the local expression of cytochromes and transporters involved in hepatic drug metabolism, impacting the global pattern of the drug pharmacokinetics both in animal and in human models (Wójcicki et al., 1999; Wang et al., 2019). Therefore, we could speculate that the altered hepatic function, underlying the increased lipid profiles, associates with individual variations of the drug bio-availability and, in turn, with the occurrence of ADR as recently observed in human models (Muirhead et al., 2002; Gao et al., 2015). In the present study, serum sampling was performed only at the patient’s recruitment and prior to any prescription of the drug, thus it was impossible to retrieve any data of sildenafil concentration from available serum samples. Further studies will warrant the validation of this model through the quantification of serum levels of sildenafil in patients with altered metabolic phenotype. Importantly, PDE5i have well-defined pharmacodynamics and are prescribed for the use on demand (Hackett, 2005). This reduces any possible bias resulting from the mutual influence of the drug activity on the metabolic phenotype of patient.

The treatment of ED represents a peculiar case of a pharmacological success. The rising incidence of metabolic diseases together with the growing base of the geriatric population, both representing important risk factors for ED, strongly nourished drug demand from patients. As a result, the market size for ED drugs in 2014 accounted for more than 4.39 billion USD (Grand View Research, 2016). However, because of the aforementioned unfavorable efficacy/safety profile, patients frequently chose to prematurely discontinue or switch to other drugs for ED, with unnecessary resource utilization (Harnett et al., 2006). Since ED burdens a severe impact on the quality of life and interpersonal relationships, the proper tailoring of its treatment has been claimed as a primary health issue (Mulhall et al., 2005; Corona et al., 2011; Corona et al., 2016). In this context, our results suggest the evaluation of serum lipid profile as a work-up procedure to be added in the clinical evaluation of ED patients. This would reasonably help the identification of patients at risk of ADR and likely addresses toward lower dosages of Sildenafil or drive the choice toward other PDE5i.

Although the small sample size of the study cohort and the exclusive inclusion of Sildenafil as PDE5i represent limitations of this study, there are nonetheless some strengths to underline. These are the thorough clinical characterization of patients and the independent evaluation of both the genetic and phenotypic profile through two new high-throughput techniques. To the best of our knowledge, this renders the present study unprecedented.

In conclusion, by the use of an integrated evaluation comprising the clinical phenotype and the genomic/metabolic profiling of ED patients, in this study we provide evidence that the occurrence of ADR associated with the use of PDE5i is strictly linked to the serum lipid pattern, in addition to the known side inhibition effect on other phosphodiesterase. These elements can help clinicians in the tailoring of individual therapies for ED patients, corroborating the favorable effect of personalized medicine.

## Data Availability Statement

Sequence data are available at SRA repository with the following url link. https://www.ncbi.nlm.nih.gov/bioproject/PRJNA674473.

## Ethics Statement

The studies involving human participants were reviewed and approved by Ethic Committee for Clinical Trials of the University-Hospital of Padova (Italy). The patients/participants provided their written informed consent to participate in this study.

## Author Contributions

MSR performed the analysis of genomic data and drafted the manuscript, AV performed NMR experiments and drafted the manuscript, LT critically discussed NMR data, MG, and MDRP performed patients recruitment and evaluation, GV and DT performed NGS sequencing, RP, and CF critically evaluated the manuscript, LDT conceived the study, performed the analysis of clinical data and drafted the manuscript. All authors contributed to the manuscript. All authors read and approved the final manuscript.

## Funding

This project has received funding from the European Union’s Horizon 2020 research and innovation programme under grant agreement No 654248. Alessia Vignoli is supported by an AIRC-Foundation for Cancer Research fellowship for Italy.

## Conflict of Interest

The authors declare that the research was conducted in the absence of any commercial or financial relationships that could be construed as a potential conflict of interest.
