# Supplementary material for: Evaluation of Serum/Urine Genomic and Metabolomic Profiles to Improve the Adherence to Sildenafil Therapy in Patients with Erectile Dysfunction
Source: Front Pharmacol. 2020 Dec 10;11:602369. doi: 10.3389/fphar.2020.602369 (PMC7849189; doi:10.3389/fphar.2020.602369)
Supplement: Supplementary file 1 [file table1.docx]

|  |  | **Response to PDE5i** | | **Adverse Drug Reaction** | |
| --- | --- | --- | --- | --- | --- |
| Parameter | All Patients (N=28) | Responders  (N=24) | Non-Responders  (N=4) | ADR  (N=12) | NO ADR  (N=16) |
| *Demographic/Clinical* |  |  |  |  |  |
| Age (years ± SD) | 47.6 ± 13.9 | 48.8 ± 13.25 | 40.5 ± 17.9 | 48.1 ± 10.4 | 47.2 ± 16.12 |
| Height (cm ± SD) | 175.5 ± 8.9 | 175.5 ± 9.5 | 175.5 ± 4.5 | 176.2 ± 6.7 | 175.1 ± 10.1 |
| Weight (kg ± SD) | 81.7 ± 17.3 | 81.6 ± 17.6 | 82.8 ± 18.3 | 78.7 ± 16.4 | 83.5 ± 18.0 |
| BMI (kg/m^2^ ± SD) | 26.4 ±4.7 | 26.3 ± 4.5 | 26.9 ± 5.9 | 25.2 ± 3.7 | 27.1 ± 5.2 |
| SBP (mmHg ± SD) | 133.2 ± 12.3 | 131.6 ± 11.4 | 133.9 ± 12.6 | 135.0 ± 12.9 | 132.1 ± 12.9 |
| DBP (mmHg ± SD) | 81.8 ± 6.0 | 80.9 ± 5.4 | 82.1 ± 5.9 | 82.5 ± 5.0 | 81.4 ± 6.9 |
| Glucose (mg/dL ± SD) | 102.3 ± 9.8 | 100.3 ± 8.4 | 103.1 ± 7.3 | 94.7 ± 6.8 | 106.2 ± 9.0 |
| Total Chol (mg/dL ± SD) | 185.4 ± 38.9 | 181.0 ± 38.1 | 187.9 ± 37.6 | 187.0 ± 30.2 | 184.4 ± 39.5 |
| HDL Chol (mg/dL ± SD) | 48.7 ± 13.9 | 49.5 ± 14.6 | 46.0 ± 12.5 | 46.0 ± 14.4 | 50 ± 14.8 |
| LDL Chol (mg/dL ± SD) | 143.7 ± 17.1 | 143.3 ± 25.7 | 149.9 ± 11.2 | 143.3 ± 13.2 | **133.1 ± 12.4*** |
| Triglycerides (mg/dL ± SD) | 102.1 ± 47.2 | 98.4 ± 48.9 | 108.6 ± 34.4 | 133.0 ± 38.5 | 86.7 ± 45.9 |
| Total T (nmol/L) | 16.9 ± 8.5 | 17.5 ± 7.2 | 15.8 ± 9.5 | 13.1 ± 7.2 | 18.0 ± 9.8 |
| LH (IU/mL ± SD) | 5.2 ± 3.11 | 4.0 ± 2.6 | 5.2 ± 3.4 | 4.9 ± 3.8 | 5.0 ± 2.9 |
| Prolactin (ng/mL ± SD) | 10.7 ± .3 | 10.5 ± 4.6 | 12.1 ± 2.4 | 9.8 ± 3.5 | 11.3 ± 4.9 |
| Organic ED (n/%) | 12/42.9% | 11/45.8% | 1/25% | 5/41.7% | 7/43.8% |
| Diabetes (n/%) | 7/25.0% | 7/14.8% | 0/0% | 2/16.7% | 5/31.3% |
| Dyslipidemia (n/%) | 10/35.7% | 8/33.3% | 2/50% | 3/25.0% | 7/43.8% |
| Hypogonadism (n/%) | 4/14.3 | 2/8.3% | 2/50% | 1/8.3% | 3/18.8% |
| Thyroid diseases (n/%) | 1/3.6% | 1/4.2% | 0/0% | 0/0% | 1/6.3% |
| *Concomitant Drug Therapy* |  |  |  |  |  |
| Glycemic Lowering Agents | 3/10.7% | 3/12.5% | 0/0% | 1/8.3% | 2/12.5% |
| Lipid Lowering Agents | 2/7.1% | 2/8.3% | 0/0% | 1/8.3% | 1/6.3% |
| Antihypertension Agents | 5/17.9% | 5/20.8% | 0/0% | 0/0% | 5/31.3% |
| Antiplatelet/anticoagulants | 3/10.7% | 3/12.5% | 0/0% | 0/0% | 3/18.75% |
| Poly-therapy | 3/10.7% | 3/12.5% | 0/0% | 0/0% | 3/18.75% |
| *Erectile Function* |  |  |  |  |  |
| IIEF15_ED_ (Score± SD) | 16.5 ± 6.8 | 17.1 ± 6.4 | 13.0 ± 8.4 | 19.5 ± 6.4 | 14.5 ± 6.4 |
| IIEF15_ORGASM_ (Score± SD) | 7.9 ± 2.5 | 7.9 ± 2.2 | 8.0 ± 4.0 | 8.2 ± 2.5 | 7.7 ± 2.5 |
| IIEF15_DESIRE_ (Score± SD) | 6.8 ± 2.1 | 7.0 ± 1.9 | 5.5 ± 2.6 | 7.5 ± 2.4 | 6.4 ± 1.8 |
| IIEF15_SATISFACTION_ (Score± SD) | 9.3 ± 4.9 | 9.2 ± 3.8 | 9.5 ± 10.3 | 12.3 ± 4.2 | 7.3 ± 4.3 |
| IIEF15_QOL_ (Score± SD) | 6.2 ± 3.9 | 6.5 ± 3.7 | 4.5 ± 5.0 | 7.3 ± 4.1 | 5.4 ± 3.7 |

**Table 1** Basal demographic and clinical parameters of patients with erectile dysfunction included in the group, distinguished for response to type 5 phosphodiesterase inhibitors and related adverse drug reactions

Abbreviations: PDE5i, type 5 phosphodiesterase inhibitors; ADR, adverse drug reaction; SD, standard deviation; BMI, body mass-index; Chol, cholesterol, HDL, High density-lipoprotein; LDL, Low density-lipoprotein; T, serum testosterone; ED, erectile dysfunction; Poly-therapy, the use of two or more different drug categories; IIEF15, 15-question International Index of Erectile Function; QOL, quality of life

Significance: *=P<0.05 *vs* ADR.
