# Supplementary material for: Evaluation of Serum/Urine Genomic and Metabolomic Profiles to Improve the Adherence to Sildenafil Therapy in Patients with Erectile Dysfunction
Source: Front Pharmacol. 2020 Dec 10;11:602369. doi: 10.3389/fphar.2020.602369 (PMC7849189; doi:10.3389/fphar.2020.602369)
Supplement: Supplementary file 2 [file table2.docx]

| **ID Gene** | **N° variants out of 2629 total variants** |
| --- | --- |
| CYP2C9 | 18 |
| CYP2D6 | 504 |
| CYP2D7 | 113 |
| CYP3A4 | 33 |
| CYP3A5 | 14 |
| GNB3 | 24 |
| LOC101929829 | 126 |
| LOC105373764 | 50 |
| NOS1 | 115 |
| NOS3 | 142 |
| PDE10A | 91 |
| PDE11A | 204 |
| PDE1A | 87 |
| PDE1B | 49 |
| PDE1C | 123 |
| PDE2A | 232 |
| PDE3A | 79 |
| PDE3B | 28 |
| PDE4A | 45 |
| PDE5A | 94 |
| PDE6A | 36 |
| PDE7A | 28 |
| PDE9A | 165 |
| VEGFA | 24 |
| ACE | 205 |
|  |  |

**Table 2** The gene panel evaluated in 28 male patients with erectile dysfunction included in the study and the corresponding number of gene variants
