# Supplementary material for: Evaluation of Serum/Urine Genomic and Metabolomic Profiles to Improve the Adherence to Sildenafil Therapy in Patients with Erectile Dysfunction
Source: Front Pharmacol. 2020 Dec 10;11:602369. doi: 10.3389/fphar.2020.602369 (PMC7849189; doi:10.3389/fphar.2020.602369)
Supplement: Supplementary file 3 [file table3.docx]

| **ID target** | **SNP** | **Responders** | **Non-Responders** | **ADR** | **NO ADR** | **Variant** |
| --- | --- | --- | --- | --- | --- | --- |
| PDE2A | rs1980091 |  |  | P=0.04; RR=2.7 [0.9-5.0] |  | Synonymous Variant |
| PDE2A | rs392565 |  |  | P=0.02; RR=3.2 [1.1-4.6] |  | Synonymous Variant |
| PDE2A | rs426907 |  |  | P=0.02; RR=3.2 [1.1-4.6] |  | Intron Variant |
| PDE3A | rs7966459 |  |  | P=0.04; RR=2.7 [0.9-5.0] |  | Intron Variant |
| PDE11A | rs10201180 | P=0.04; RR=2.7 [0.9-5.1] |  |  |  | Splice region variant |
| CYP2C9 | rs1799853 |  |  |  | p=0.02.RR=0.0 [0-1.02] | Missense Variant |
| CYP2C9 | rs9332119 |  |  |  | p=0.02.RR=0.0 [0-1.02] | Intron Variant |
| CYP2D7 | rs56127449 | P=0.04; RR=2.7 [0.9-5.1] |  |  |  | Missense Variant |

**Table 3** Differential clustering of gene variants detected in 28 male patients with erectile dysfunction included in the study, grouped according to drug response of presence of adverse drug reaction.

Abbreviations: DR=drug responsive. NDR=not drug responsive. ADR=adverse drug reactions. NADR= not adverse drug reactions. RR=Relative Risk (95% confidence intervals are reported within squared brackets)
