# Supplementary material for: Evaluation of Serum/Urine Genomic and Metabolomic Profiles to Improve the Adherence to Sildenafil Therapy in Patients with Erectile Dysfunction
Source: Front Pharmacol. 2020 Dec 10;11:602369. doi: 10.3389/fphar.2020.602369 (PMC7849189; doi:10.3389/fphar.2020.602369)
Supplement: Supplementary file 4 [file table4.docx]

**Table 4**. Lipid analysis in serum samples from the 26 out of 28 patients with ED included in the metabolomics analysis. A positive Log2(FC) value means higher level in patients that experienced adverse drug reactions.

| **Lipid Fraction** | ***p*-value** | **AUROC** | **Log2(FC)** |
| --- | --- | --- | --- |
| **Triglycerides (TG)** | **0.019** | **0.793** | **0.422** |
| **Cholesterol (Chol)** | **0.019** | **0.793** | **0.417** |
| **LDL Cholesterol (LDL Chol)** | **0.028** | **0.777** | **0.541** |
| HDL Cholesterol (HDL Chol) | 0.401 | 0.612 | 0.163 |
| Apo A1 | 0.193 | 0.669 | 0.193 |
| Apo A2 | 0.212 | 0.661 | 0.294 |
| **Apo B100** | **0.040** | **0.760** | **0.480** |
| **Lipoprotein Main Fractions Triglycerides VLDL** | **0.007** | **0.835** | **0.675** |
| **Lipoprotein Main Fractions Triglycerides IDL** | **0.023** | **0.785** | **0.797** |
| Lipoprotein Main Fractions Triglycerides LDL | 0.108 | 0.707 | 0.349 |
| Lipoprotein Main Fractions Triglycerides HDL | 0.974 | 0.508 | 0.005 |
| **Lipoprotein Main Fractions Cholesterol VLDL** | **0.023** | **0.785** | **0.590** |
| **Lipoprotein Main Fractions Cholesterol IDL** | **0.023** | **0.785** | **0.912** |
| **Lipoprotein Main Fractions Cholesterol LDL** | **0.028** | **0.777** | **0.541** |
| Lipoprotein Main Fractions Cholesterol HDL | 0.401 | 0.612 | 0.163 |
| **Lipoprotein Main Fractions Free Cholesterol VLDL** | **0.016** | **0.802** | **0.512** |
| **Lipoprotein Main Fractions Free Cholesterol IDL** | **0.013** | **0.810** | **0.951** |
| **Lipoprotein Main Fractions Free Cholesterol LDL** | **0.019** | **0.793** | **0.443** |
| Lipoprotein Main Fractions Free Cholesterol HDL | 0.699 | 0.554 | 0.089 |
| **Lipoprotein Main Fractions Phospholipids VLDL** | **0.019** | **0.793** | **0.594** |
| **Lipoprotein Main Fractions Phospholipids IDL** | **0.015** | **0.810** | **0.927** |
| **Lipoprotein Main Fractions Phospholipids LDL** | **0.034** | **0.769** | **0.450** |
| Lipoprotein Main Fractions Phospholipids HDL | 0.606 | 0.570 | 0.099 |
| Lipoprotein Main Fractions Apo A1 HDL | 0.243 | 0.653 | 0.180 |
| Lipoprotein Main Fractions Apo A2 HDL | 0.243 | 0.653 | 0.280 |
| **Lipoprotein Main Fractions Apo B VLDL** | **0.034** | **0.769** | **0.537** |
| **Lipoprotein Main Fractions Apo B IDL** | **0.023** | **0.785** | **0.742** |
| **Lipoprotein Main Fractions Apo B LDL** | **0.047** | **0.752** | **0.481** |
| **VLDL Subfractions Triglycerides VLDL 1** | **0.016** | **0.802** | **0.566** |
| **VLDL Subfractions Triglycerides VLDL 2** | **0.007** | **0.843** | **0.866** |
| **VLDL Subfractions Triglycerides VLDL 3** | **0.004** | **0.851** | **0.980** |
| **VLDL Subfractions Triglycerides VLDL 4** | **0.007** | **0.835** | **0.833** |
| VLDL Subfractions Triglycerides VLDL 5 | 0.797 | 0.537 | 0.082 |
| VLDL Subfractions Cholesterol VLDL 1 | 0.438 | 0.603 | 0.264 |
| **VLDL Subfractions Cholesterol VLDL 2** | **0.022** | **0.793** | **0.677** |
| **VLDL Subfractions Cholesterol VLDL 3** | **0.006** | **0.851** | **1.068** |
| **VLDL Subfractions Cholesterol VLDL 4** | **0.019** | **0.793** | **0.889** |
| VLDL Subfractions Cholesterol VLDL 5 | 0.768 | 0.541 | 0.048 |
| VLDL Subfractions Free Cholesterol VLDL 1 | 0.088 | 0.719 | 0.721 |
| **VLDL Subfractions Free Cholesterol VLDL 2** | **0.019** | **0.793** | **0.741** |
| **VLDL Subfractions Free Cholesterol VLDL 3** | **0.009** | **0.835** | **1.058** |
| **VLDL Subfractions Free Cholesterol VLDL 4** | **0.013** | **0.818** | **1.040** |
| VLDL Subfractions Free Cholesterol VLDL 5 | 0.554 | 0.579 | -0.174 |
| VLDL Subfractions Phospholipids VLDL 1 | 0.047 | 0.752 | 0.605 |
| **VLDL Subfractions Phospholipids VLDL 2** | **0.005** | **0.843** | **0.803** |
| **VLDL Subfractions Phospholipids VLDL 3** | **0.003** | **0.876** | **1.012** |
| **VLDL Subfractions Phospholipids VLDL 4** | **0.017** | **0.806** | **0.720** |
| VLDL Subfractions Phospholipids VLDL 5 | 0.430 | 0.603 | 0.134 |
| LDL Subfractions Triglycerides LDL 1 | 0.652 | 0.562 | 0.114 |
| **LDL Subfractions Triglycerides LDL 2** | **0.040** | **0.760** | **0.319** |
| LDL Subfractions Triglycerides LDL 3 | 0.622 | 0.566 | 0.147 |
| LDL Subfractions Triglycerides LDL 4 | 0.101 | 0.711 | 0.395 |
| **LDL Subfractions Triglycerides LDL 5** | **0.033** | **0.773** | **0.534** |
| **LDL Subfractions Triglycerides LDL 6** | **0.049** | **0.752** | **0.354** |
| LDL Subfractions Cholesterol LDL 1 | 0.300 | 0.636 | 0.261 |
| LDL Subfractions Cholesterol LDL 2 | 0.519 | 0.587 | 0.257 |
| LDL Subfractions Cholesterol LDL 3 | 0.065 | 0.736 | 0.481 |
| **LDL Subfractions Cholesterol LDL 4** | **0.019** | **0.793** | **0.590** |
| **LDL Subfractions Cholesterol LDL 5** | **0.028** | **0.777** | **0.711** |
| **LDL Subfractions Cholesterol LDL 6** | **0.040** | **0.760** | **0.582** |
| LDL Subfractions Free Cholesterol LDL 1 | 0.057 | 0.744 | 0.399 |
| LDL Subfractions Free Cholesterol LDL 2 | 0.171 | 0.678 | 0.424 |
| **LDL Subfractions Free Cholesterol LDL 3** | **0.040** | **0.760** | **0.361** |
| **LDL Subfractions Free Cholesterol LDL 4** | **0.034** | **0.769** | **0.529** |
| **LDL Subfractions Free Cholesterol LDL 5** | **0.040** | **0.760** | **0.619** |
| **LDL Subfractions Free Cholesterol LDL 6** | **0.047** | **0.752** | **0.593** |
| LDL Subfractions Phospholipids LDL 1 | 0.401 | 0.612 | 0.190 |
| LDL Subfractions Phospholipids LDL 2 | 0.562 | 0.579 | 0.225 |
| LDL Subfractions Phospholipids LDL 3 | 0.065 | 0.736 | 0.398 |
| **LDL Subfractions Phospholipids LDL 4** | **0.019** | **0.793** | **0.520** |
| **LDL Subfractions Phospholipids LDL 5** | **0.023** | **0.785** | **0.654** |
| **LDL Subfractions Phospholipids LDL 6** | **0.034** | **0.769** | **0.518** |
| LDL Subfractions Apo B LDL 1 | 0.293 | 0.636 | 0.203 |
| LDL Subfractions Apo B LDL 2 | 0.401 | 0.612 | 0.253 |
| **LDL Subfractions Apo B LDL 3** | **0.040** | **0.760** | **0.427** |
| **LDL Subfractions Apo B LDL 4** | **0.019** | **0.793** | **0.576** |
| **LDL Subfractions Apo B LDL 5** | **0.028** | **0.777** | **0.691** |
| **LDL Subfractions Apo B LDL 6** | **0.047** | **0.752** | **0.522** |
| HDL Subfractions Triglycerides HDL 1 | 0.470 | 0.595 | -0.311 |
| HDL Subfractions Triglycerides HDL 2 | 0.652 | 0.562 | -0.067 |
| HDL Subfractions Triglycerides HDL 3 | 0.431 | 0.603 | 0.159 |
| HDL Subfractions Triglycerides HDL 4 | 0.148 | 0.686 | 0.202 |
| HDL Subfractions Cholesterol HDL 1 | 0.847 | 0.529 | 0.005 |
| HDL Subfractions Cholesterol HDL 2 | 0.844 | 0.529 | 0.108 |
| HDL Subfractions Cholesterol HDL 3 | 0.438 | 0.603 | 0.221 |
| HDL Subfractions Cholesterol HDL 4 | 0.101 | 0.711 | 0.270 |
| HDL Subfractions Free Cholesterol HDL 1 | 0.949 | 0.512 | 0.148 |
| HDL Subfractions Free Cholesterol HDL 2 | 0.606 | 0.570 | 0.223 |
| HDL Subfractions Free Cholesterol HDL 3 | 0.332 | 0.628 | 0.403 |
| HDL Subfractions Free Cholesterol HDL 4 | 0.082 | 0.723 | 0.352 |
| HDL Subfractions Phospholipids HDL 1 | 0.478 | 0.595 | -0.153 |
| HDL Subfractions Phospholipids HDL 2 | 0.949 | 0.512 | 0.043 |
| HDL Subfractions Phospholipids HDL 3 | 0.669 | 0.558 | 0.193 |
| HDL Subfractions Phospholipids HDL 4 | 0.171 | 0.678 | 0.215 |
| HDL Subfractions Apo A1 HDL 1 | 0.606 | 0.570 | -0.194 |
| HDL Subfractions Apo A1 HDL 2 | 0.797 | 0.537 | 0.100 |
| HDL Subfractions Apo A1 HDL 3 | 0.438 | 0.603 | 0.176 |
| HDL Subfractions Apo A1 HDL 4 | 0.076 | 0.727 | 0.247 |
| HDL Subfractions Apo A2 HDL 1 | 0.844 | 0.529 | 0.073 |
| HDL Subfractions Apo A2 HDL 2 | 0.699 | 0.554 | 0.328 |
| HDL Subfractions Apo A2 HDL 3 | 0.401 | 0.612 | 0.344 |
| **HDL Subfractions Apo A2 HDL 4** | **0.040** | **0.760** | **0.300** |

Abbreviations: AUROC, Area under the receiver operating characteristics curve;
