# Supplementary material for: Evaluation of Serum/Urine Genomic and Metabolomic Profiles to Improve the Adherence to Sildenafil Therapy in Patients with Erectile Dysfunction
Source: Front Pharmacol. 2020 Dec 10;11:602369. doi: 10.3389/fphar.2020.602369 (PMC7849189; doi:10.3389/fphar.2020.602369)
Supplement: Supplementary file 5 [file table5.docx]

**Table 5.** Serum metabolite univariate analysis. A positive Log2(FC) means higher level in patients that experienced adverse effects.

|  | *P*-value | AUROC | Log2(FC) |
| --- | --- | --- | --- |
| Alanine | 0.308 | 0.632 | 0.077 |
| Creatinine | 0.687 | 0.554 | 0.109 |
| Glutamic acid | 0.528 | 0.579 | 0.145 |
| Glutamine | 0.375 | 0.616 | 0.133 |
| Glycine | 0.717 | 0.550 | 0.064 |
| Histidine | 0.344 | 0.620 | 0.144 |
| Isoleucine | 0.612 | 0.566 | 0.065 |
| Leucine | 0.146 | 0.686 | 0.241 |
| Lysine | 0.817 | 0.533 | -0.199 |
| Ornithine | 0.573 | 0.574 | 0.090 |
| Phenylalanine | 0.402 | 0.607 | 0.215 |
| **Threonine** | **0.009** | **0.831** | **0.621** |
| Tyrosine | 0.893 | 0.521 | 0.022 |
| Valine | 1.000 | 0.504 | 0.040 |
| Acetic acid | 0.916 | 0.517 | -0.038 |
| Citric acid | 0.895 | 0.521 | 0.074 |
| Formic acid | 0.503 | 0.587 | 0.135 |
| Lactic acid | 0.393 | 0.612 | 0.020 |
| 3-Hydroxybutyric acid | 0.099 | 0.702 | -1.728 |
| Acetone | 0.588 | 0.570 | 0.142 |
| Pyruvic acid | 0.387 | 0.612 | -0.096 |
| Glucose | 0.278 | 0.640 | 0.145 |
