# Supplementary material for: Evaluation of Serum/Urine Genomic and Metabolomic Profiles to Improve the Adherence to Sildenafil Therapy in Patients with Erectile Dysfunction
Source: Front Pharmacol. 2020 Dec 10;11:602369. doi: 10.3389/fphar.2020.602369 (PMC7849189; doi:10.3389/fphar.2020.602369)
Supplement: Supplementary file 6 [file table6.docx]

**Table 6.** Metabolite analysis in urine samples. A positive Log2(FC) means higher level in patients that experienced adverse effects.

|  | *P*-value | AUROC | Log2(FC) |
| --- | --- | --- | --- |
| Acetic acid | 0.671 | 0.548 | 0.940 |
| Acetoacetic acid | 0.448 | 0.557 | -1.339 |
| Acetone | 0.098 | 0.685 | -1.030 |
| Alanine | 0.865 | 0.523 | 0.032 |
| Betaine | 0.604 | 0.563 | -0.202 |
| Citric acid | 0.368 | 0.608 | 0.218 |
| Creatinine | 0.481 | 0.585 | 0.064 |
| D-Glucose | 0.249 | 0.605 | 1.042 |
| Formic acid | 0.276 | 0.625 | -0.428 |
| Glycine | 0.336 | 0.614 | -0.242 |
| Hippuric acid | 0.486 | 0.580 | 0.127 |
| Methanol | 0.535 | 0.571 | -0.099 |
| Methylmalonic acid | 0.812 | 0.523 | 0.113 |
| N-Isovaleroylglycine | 0.812 | 0.523 | 0.216 |
| N-N-Dimethylglycine | 0.297 | 0.622 | -0.739 |
| Oxaloacetic acid | 0.059 | 0.696 | -1.742 |
| Oxypurinol | 0.120 | 0.614 | 3.862 |
| Proline betaine | 0.520 | 0.557 | -1.698 |
| Propylene glycol | 0.497 | 0.551 | -2.066 |
| Succinic acid | 0.110 | 0.676 | -0.968 |
| Tartaric acid | 0.314 | 0.614 | -1.097 |
| Trigonelline | 0.974 | 0.506 | -0.048 |
| Uracil | 0.480 | 0.580 | 0.467 |
| Valine | 0.361 | 0.608 | 0.325 |
