# Supplementary material for: Evaluation of Serum/Urine Genomic and Metabolomic Profiles to Improve the Adherence to Sildenafil Therapy in Patients with Erectile Dysfunction
Source: Front Pharmacol. 2020 Dec 10;11:602369. doi: 10.3389/fphar.2020.602369 (PMC7849189; doi:10.3389/fphar.2020.602369)
Supplement: Supplementary file 7 [file table7.docx]

**Table 7**. Association between the main lipid fractions, obtained by metabolomics analysis, and the occurrence of adverse drug reactions in 26 out of 28 patients with ED included in the analysis. Risk threshold for each lipid fractions were arbitrarily set at each median value.

| **Main Lipid Fraction** | **HR** | ***p*-value** |
| --- | --- | --- |
| **Triglycerides, TG ≥ 80** | **7.111** | **0.040** |
| **Cholesterol, Chol ≥ 190** | **7.111** | **0.040** |
| **LDL Cholesterol, LDL Chol ≥ 100** | **17.500** | **0.019** |
| HDL Cholesterol, HDL Chol ≥ 50 | 0.686 | 0.665 |
| Apo A1, Apo A1 ≥ 140 | 1.458 | 0.665 |
| Apo A2, Apo A2 ≥ 30 | 1.458 | 0.665 |
| Apo B100, Apo B100 ≥ 80 | 3.200 | 0.200 |

Abbreviations: HR, hazard ratio
